# Pandemic influenza – Indian experience

**DOI:** 10.4103/0970-2113.76292

**Published:** 2011

**Authors:** J. C. Suri, M. K. Sen

**Affiliations:** *Department of Pulmonary, Critical Care and Sleep Medicine, New Delhi, India. E-mail: jcsuri@rediffmail.com*

**Figure 1:**
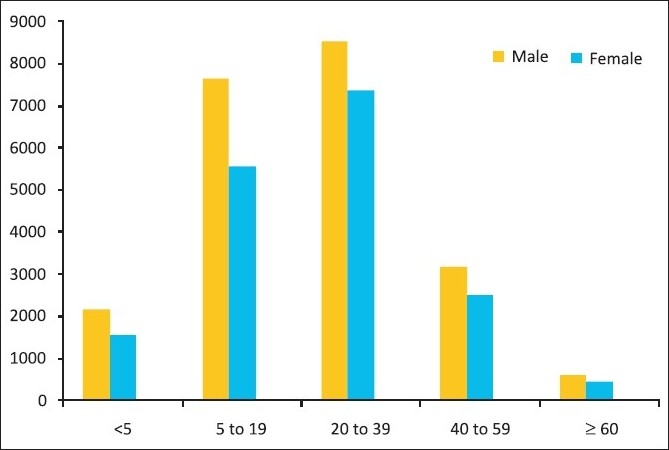
Age and sex pattern of recoded cases, Data Source: Ministry of Health and Family Welfare, Govt. of India

Pandemic influenza (also termed H1N1 influenza/novel influenza/“Swine flu”) has ravaged the health scenario worldwide.[1–10] Resource limited countries have not been an exception. The occurrence of a large number of such cases in India has allowed us to draw our own conclusions about issues related to handling the situation within the framework of international guidelines. A review of weekly trends in the prevalence of the disease reveals that there have been peaks in the prevalence of the disease in August–September and November–December, 2009. An almost similar increase in the number of such cases has been noticed in 2010 as well. The gender-wise distribution of cases has shown a male predominance[11] [Figure 1].

The containment of the pandemic in India was possible only by the prompt and efficient measures taken up by the Government of India. Immediately after cases were reported in Pune, Maharashtra, and other places all over the country, steps were taken to chalk out guidelines for control of the disease in India. Isolation of the cases in the healthcare facilities and home environment (depending on severity of illness), categorization of cases, providing treatment to suitable individuals (cases and household contacts), public education, and other appropriate measures have proved very effective in due course of time.

Healthcare authorities in India have developed a strategy of categorization of patients [Table 1] in three major groups in order to channelize available diagnostic and therapeutic measures to those who had the highest probability of manifesting severe forms of the disease.[11]

**Table 1:** Clinical categorization of patients

Category A
i) Patients with mild fever plus cough / sore throat with or without body ache, headache, diarrhea, and vomiting will be categorized as Category A. They do not require Oseltamivir and should be treated for the symptoms mentioned above. The patients should be monitored for their progress and reassessed at 24 to 48 h by the doctor. ii) No testing of the patient for H1N1 is required. iii) Patients should confine themselves at home and avoid mixing with public and high-risk members in the family.
Category B
i) In addition to all the signs and symptoms mentioned under Category A, if the patient has high grade fever and severe sore throat, may require home isolation and Oseltamivir; ii) In addition to all the signs and symptoms mentioned under Category A, individuals having one or more of the following high-risk conditions shall be treated with Oseltamivir: Children with mild illness but with predisposing risk factors; Pregnant women; Persons aged 65 years or older; Patients with lung diseases, heart disease, liver disease, kidney disease, blood disorders, diabetes, neurological disorders, cancer, and HIV/AIDS; Patients on long-term cortisone therapy. No tests for H1N1 are required for Category B (i) and (ii). All patients of Category B (i) and (ii) should confine themselves at home and avoid mixing with public and high-risk members in the family.
Category C
i) In addition to the above signs and symptoms of Category A and B, if the patient has one or more of the following: Breathlessness, chest pain, drowsiness, fall in blood pressure, sputum mixed with blood, bluish discoloration of nails; Children with influenza like illness who had a severe disease as manifested by the red flag signs (somnolence, high and persistent fever, inability to feed well, convulsions, shortness of breath, difficulty in breathing, etc). Worsening of underlying chronic conditions. All these patients mentioned above in Category C require testing, immediate hospitalization, and treatment.

**Figure 2:**
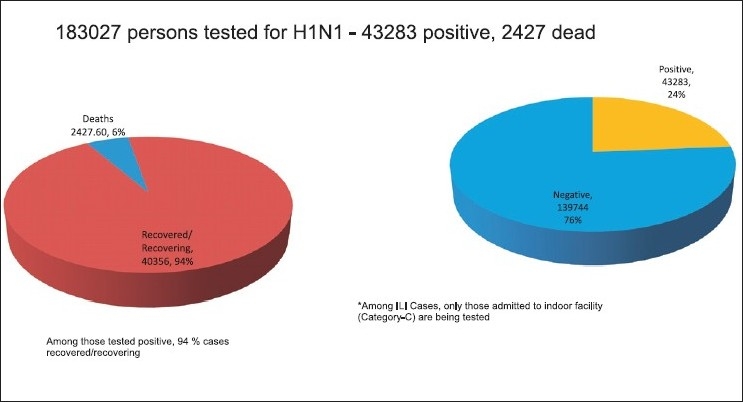
Magnitude of H1N1 problem, Data Source: Ministry of Health and Family Welfare, Govt. of India

Laboratory testing, under this strategy, was conducted in patients belonging to Category-C; treatment with anti-viral drug Oseltamivir was, however, restricted to severe cases only, even if they were not lab tested. Among those tested, 94% of cases were found to have recovered[11] [Figure 2]. In addition to Govt. labs, several privately owned laboratories were also accredited for H1N1 testing.

A valid observation that is made from the data on laboratory testing is that a large majority of those individuals who were tested (nasal/oral swabs for RT-PCR) have been declared negative for the virus [Figure 2]. One of the attributing factors for this fact might have been the concurrent occurrence of other viral diseases like dengue, malaria, and other respiratory viral infections. Thus, a large number of patients seem to have been tested over enthusiastically. In future, it may be prudent to limit the use of such an expensive laboratory testing only to those who are most likely to harbor the disease. This would also effectively prevent the overwhelming of the limited laboratory resources. Laboratories, in turn, can process the samples faster and furnish reports to the clinician earlier.

Several factors were observed to be associated with increased morbidity and mortality. These can be broadly grouped under four categories: those pertaining to (a) diagnostic factors (b) evaluation of severity of illness (c) management issues, and (d) delivery of care.

Issues pertaining to diagnosis were mainly clustered around the following factors: (i) lack of access to health care – mainly in remotely situated pockets in the interior regions; (ii) seeking care from traditional healers like village doctors/registered medical practitioners; and (iii) varied clinical picture of the disease (for instance a high mortality was observed in pregnancy and a rapid progression of disease from influenza-like illness (ILI) to acute lung injury and acute respiratory distress syndrome in some individuals); (iv) the high prevalence of confounding and/or co-infecting illnesses like malaria, dengue, chikungunya and other viral illness often vitiates the clinical picture and creates a diagnostic confusion. Viral co-infection demands testing for multiple viruses and may be associated with an increase in morbidity and mortality; (v) a large prevalence of associated chronic illnesses like tuberculosis, kala-azar, malnutrition, etc. Such illnesses may predispose the patient to acquire ILI.

Another impediment to early diagnosis is the lack of any rapid, point-of-care diagnostic tool. The rapid influenza diagnostic tests (RIDTs) have sensitivity between 20 and 90% as compared to RT-PCR and vial culture. The sensitivity depends on age, duration of illness, sample type, and viral strain.

The various factors pertaining to poor assessment of severity of illness are as follows. Firstly there is largely a poor assessment of cases at the primary care level. Innumerable cases of delayed referral to higher centers, resulting from delayed recognition of acute hypoxemic respiratory failure (ALI-ARDS) have been observed. A possible solution may include proper training of healthcare personnel at primary healthcare centers/sub-centers, community health centers, and district hospitals. Provision of simple and cheap monitoring devices like finger-probe oximeters may also be considered as a useful measure to overcome non-availability of simple tools to assess oxygenation status. A lack of clinical and laboratory markers which can predict serious forms of disease, particularly in patients without co-morbidity, is also a handicap. It may be added that unlike many other illnesses, there is no validated, severity assessment score, specific to influenza that is available to clinicians till date.

All these factors may often lead to a delay in referral to the hospital by peripheral centers, resulting in late initiation of treatment and higher mortality.

There are several issues relating to drug therapy that need to be considered. Antiviral therapy with Oseltamivir was given only to all high-risk and seriously ill patients under the Govt. of India strategy. In addition, prophylactic treatment was given to high-risk contacts of all seropositive patients. Not treating patients with mild disease did not seem to have any adverse impact on the outcome of the epidemic in India. It is also noteworthy that due to non-availability of intravenous preparations of Oseltamivir and other drugs, difficulties were often faced in treating patients on ventilator who had a non-functional gut.

In the absence of clear-cut guidelines with regard to maximal drug dose, maximal prolongation in duration of treatment and combination of multiple of anti-viral drugs etc, extensive variability in management patterns have been observed amongst physicians.

As far as healthcare delivery is concerned, it has been observed that there may have been cases of delay, particularly in referral of cases to higher centers and institution of specific supportive treatment. As mentioned earlier, lack of adequately trained manpower may be a contributing factor. Also, there is a dirth of adequate number of high-dependency units and intensive care units, specially dedicated to management of pandemic influenza. This is particularly so in the peripheral and interiorly located regions. A large number of isolation ICUs were created in major hospitals at the initiative of Ministry of Health and Family Welfare, Govt. of India. These units have successfully managed seriously ill influenza patients. Fortunately, the overall severity of the epidemic was generally mild this time. However, if the next wave of the epidemic is more severe, there would be a need to significantly enhance the availability of ICU and HDU beds.

The socio-culture milieu in our country offers a perfect setting for the re-assortment of the pandemic influenza virus. Rural India has the human host, poultry birds, and the pigs sharing the same premises for habitation in close proximity. As predicted by public health experts, the scourge of the pandemic is far from being over. In such circumstances, developing countries like India would, therefore, be fertile grounds to bear the brunt, in case of resurgence of the disease.

In the backdrop of the aforementioned facts, some remedial measurements may always be suggested. Development of community-based modules must aim at optimizing treatment at the primary care level with a focus on early evaluation of severity of illness, provision of early and effective treatment so as to bring about a decrease in morbidity and mortality. Workshops may be arranged to train primary care physicians in early diagnosis, endotracheal intubation, oxygen therapy, and non-invasive ventilation. High-dependency units may be installed and made functional at district-level hospitals. Mobile ICUs may be provided and access to expert critical care physicians be extended by telemedicine, telephone hotlines, and Internet. Websites and webcasts to disseminate knowledge about pandemic influenza cases may be very useful.

With the vast data at our disposal, several areas of research can be identified. Studies to find out the clinical profile, severity, complications, and the co-morbidities are required. Based on the audit of hospitalized patients, clinical and biochemical markers which can predict the development of serious disease should be studied. Particular mention is made about issues related to development of intravenous preparation of anti-H1N1 influenza drugs, studies to assess the ideal infection control measures in ICU (role of different types of cost-effective masks), bronchoalveolar lavage fluid, lung biopsy specimens, and post-mortem specimens in the understanding of pathophysiology and pathogenesis of the disease and further genetic research for identifying high-risk groups. This issue of Lung India contains articles covering some of these aspects.[12–14]
